# The Role of Intestinal Microbiota in Celiac Disease and Further Therapeutic Perspectives

**DOI:** 10.3390/life13102039

**Published:** 2023-10-11

**Authors:** Oana Belei, Iulius Jugănaru, Diana-Georgiana Basaca, Andrei Ioan Munteanu, Otilia Mărginean

**Affiliations:** 1 First Pediatric Clinic, Disturbances of Growth and Development on Children Research Center, “Victor Babeș” University of Medicine and Pharmacy, 300041 Timișoara, Romania; belei.oana@umft.ro (O.B.); diana.basaca@umft.ro (D.-G.B.); andrei-ioan.munteanu@umft.ro (A.I.M.); marginean.otilia@umft.ro (O.M.); 2 First Pediatric Clinic, “Victor Babeș” University of Medicine and Pharmacy, 300041 Timișoara, Romania

**Keywords:** celiac disease, intestinal microbiota, gluten-free diet

## Abstract

Celiac disease (CD) is an immune-mediated enteropathy caused by exposure to gluten and related prolamins in genetically susceptible individuals. It is a complex genetic disorder with multiple contributing genes. Linkage studies have identified several genomic regions that probably contain CD susceptibility genes. The most important genetic factors are HLA-DQ2 and DQ8. Several known environmental triggers promote the onset of CD at any age after gluten introduction in individuals with a genetic background, such as viral infections and intestinal dysbiosis. Recent publications have described the interference of the intestinal microbiome in gluten metabolism, modulation of local immune reactions, and in maintaining normal gut permeability. These results have promoted further lines of research on the benefit of probiotic administration to prevent disease onset or alleviate clinical symptoms along with a gluten-free diet (GFD). The relationship between gut microbiome changes and the onset of CD is incompletely understood, still being the subject of current research. This narrative review analyzes the interplay between environmental factors, intestinal microbiome alterations, and the course of CD. Furthermore, this review sets out to discuss if modulation of intestinal microflora with pre- and probiotics along with a GFD could represent a reliable therapeutic target for celiac patients.

## 1. Introduction

The most extensively used food grain worldwide is wheat. The primary complex of structural proteins in wheat is called gluten, with gliadins and glutenins being two of the potential nocive components for subjects with a particular genetic background. The phrase “gluten-related disorders” is a catch-all phrase used to refer to ailments brought on by eating things containing gluten [1]. When certain persons with the DQ2 and/or DQ8 HLA gene are exposed to dietary gluten and related prolamins, they develop celiac disease (CD), an immune-mediated enteropathy. It affects both symptomatic and asymptomatic people, including those with type I diabetes, autoimmune diseases, immunoglobulin A (Ig A) deficiency, and first-degree relatives of people with CD. Studies on the atypical or silent type of gluten enteropathy have sparked a lot of interest in serological screening techniques for CD diagnosis. In clinical practice, CD serological testing is helpful in identifying patients who need intestinal biopsies to confirm the diagnosis. Anti-endomysium (EMA) and anti-tissue transglutaminase 2 (anti-tTG2) autoantibodies are particular markers for CD. Both tests are extremely sensitive and specific, with values for both parameters typically reaching 96% [2].

An unfavorable immunologic response to wheat proteins is known as wheat allergy (WA). The gastrointestinal mucosa’s T cells are what cause the sensitivity to gluten in both cases. Wheat-specific Ig E antibodies are essential in the pathophysiology of WA because they cause the release of chemical mediators such as histamine from mast cells and basophils [3]. Contrarily, the specific serologic autoantibodies tTG and EMA show that CD is an autoimmune illness. There are instances of gluten reactions outside of CD and WA where neither autoimmune nor allergic processes are at play, commonly referred to as non-celiac gluten sensitivity (NCGS). Diagnosed by exclusion criteria, NCGS refers to gluten reactions in which both allergy and autoimmune causes have been ruled out [4].

According to epidemiological studies conducted in Europe and the United States, between 0.5 and 1% of the general population has CD. According to reports, the prevalence of CD in children ranges from 1:285 to 1:77 in Sweden, from 1:99 to 1:67 in Finland, and from 1:230 to 1:106 in school-aged Italian students. Adults with small intestinal biopsy-confirmed CD are thought to occur between 2 and 13/100,000 times each year, according to population-based estimations [5].

Multiple genes contribute to CD, a complicated genetic condition. Several genomic areas that most likely include CD susceptibility genes have been found through linkage studies. HLA-DQ2 and HLA-DQ8, which are required but insufficient to predispose to gluten enteropathy, have been identified as the most significant genetic factors. Non-HLA genome-wide linkage and association studies have identified substantially weaker relationships. This may be due to the fact that numerous non-HLA genes are involved in the etiology of CD. Consequently, the effect of a single non-HLA predisposing gene may not be very significant. Practically all celiac disease sufferers have HLA-DQ2 or HLA-DQ8, and the absence of these components has a CD negative predictive value of nearly 100% [6]. In clinical practice, genetic risk profiles for CD could be useful for predicting disease susceptibility and progression. The primary function of HLA typing is to rule out this disorder according to its significant negative predictive value. When individuals are at high risk for developing CD in the future, HLA typing can assist with ruling out this possibility and provide further information if the clinical picture is ambiguous [6].

Several known environmental triggers promote the onset of CD at any age after gluten introduction in individuals with genetic backgrounds (HLA-DQ2/DQ8 positivity and non-HLA loci), such as viral infections and intestinal dysbiosis [6].

Recent publications have described the interference of the intestinal microbiome in gluten metabolism, modulation of local immune reactions, and in maintaining the gut barrier’s normal permeability. These results have promoted further lines of research on the benefit of probiotic administration in CD patients in order to prevent disease onset or alleviate clinical symptoms along with a GFD [7,8].

## 2. Research Methodology

The authors performed systematic literature research focused on intestinal microbiota alterations in CD patients and implications regarding the course of the disease. This research was conducted by searching PubMed, Scopus, Medline, and Cochrane Library databases from January 2000 to December 2022. All publications focusing on intestinal microbiota and pathogenic, clinical, diagnostic, and therapeutic interventions in CD patients were assessed. The inclusion criteria used to extract relevant information were clinical and preclinical/laboratory studies published in the English language in the last 22 years, with a sample size comprising more than 15 subjects. The most important data are summarized in this narrative review.

## 3. Objectives of the Study

This review aims to analyze the influence of intestinal microbiota modulation on the outcomes of intestinal inflammation among pediatric and adult patients with CD. The relationship between gut microbiome changes and the onset of gluten enteropathy in genetically predisposed individuals is incompletely understood, still being the subject of current research. There are a lot of factors influencing CD onset and the evolutive pattern (environmental, genetic factors, intestinal microbiota imbalance, and intestinal immunity impairment). This paper aims to analyze the interplay between these factors and the course of CD. Furthermore, this review sets out to assess if modulation of intestinal microflora with pre- and probiotics along with a GFD could represent a reliable therapeutic target for CD patients.

## 4. Overview of Celiac Disease Epidemiology and Pathophysiology

### 4.1. Epidemiology

The prevalence of CD in the general population is 0.5–1% (Table 1), being one of the most common autoimmune pathologies, and this being lower in populations with a low gluten diet [9,10,11,12,13,14,15]. The varied symptomatology and/or the lack of knowledge of pathology, in the absence of serological screening programs, reduce the chances of diagnosis, as it emerges from specialized studies.

In developed countries, the number of cases is increasing, as evidenced by the fact that in the USA, the number of cases increased five times between 1975 and 2000, without an explanation until now [16]. First-degree relatives (10–15%) as well as patients with Down syndrome, type 1 diabetes, or IgA deficiency are more at risk [17].

CD is more common in girls and can occur after gluten introduction at any age, including infancy [15,18,19,20,21]. Prevalence by sex and age group shows a 1.5- to 2-fold higher risk in women [6,15,19,20,21], and it is higher in children than in adults (0.9% versus 0.5%) [15]. The cumulative prevalence of CD from 1991 to 2000 was 0.6%, and from 2011 to 2016 it was 0.8%. These results show an increase in the prevalence of the disease [15]. The advent of non-invasive and accurate serological tests has led to an increase in the number of cases diagnosed with CD [22]. For example, in Canada, the number of cases in children increased threefold after the introduction of EMA testing [23]. Even though the diagnostic modality and practice guidelines have been updated in parallel with the increase in awareness and the level of information about CD, up to 95% of patients with this pathology remain undiagnosed [24,25]. Some studies have found that the delay in diagnosis ranges from 4 to 10 years [26,27].

Because of nonspecific symptoms or mild manifestations, the diagnosis is missed even in developed countries [28]. Table 2 shows the global prevalence of CD. Of course, the outcome is even worse in underdeveloped countries, a consequence of reduced access to diagnostic tests and lack of experience [29].

In Japan, the number of cases is low, with the low prevalence causing general practitioners not to address CD in at-risk patients; therefore, physicians in this country must perform specific tests for CD in the high-risk population [31].

### 4.2. Pathophysiology

The uniqueness of CD stems from the fact that its major genetic components (human leukocyte antigen (HLA)-DQ2 and HLA-DQ8), the autoantigen involved (tissue transglutaminase (tTG)), and the environmental trigger (undigested immunogenic peptides of gluten) are well characterized. However, the absence of a suitable animal model has been a significant challenge in studying this disease. The Irish setter dog is the only known animal model capable of developing gluten-related pathology [32]. Nonetheless, advancements in medical and research technologies are now offering new possibilities for important breakthroughs in understanding CD.

The prevailing notion that gluten serves as the primary determinant triggering disease onset in genetically susceptible individuals has encountered skepticism in light of a notable surge in autoimmune conditions. Furthermore, heightened emphasis on cleanliness and measures aimed at shielding against microorganism exposure have shown an association with a marked escalation in autoimmune disorders within industrialized nations throughout the last four decades [17,33].

Alterations in environmental conditions and lifestyle patterns have potentially contributed to a rise in autoimmune disease prevalence by diminishing pathogen exposure. The scientific community is actively investigating the hypothesis that gut microbiota [34] plays a pivotal role in maintaining the delicate equilibrium between immune response and tolerance, thereby influencing the development of autoimmunity. While the specific impact of microorganism exposure on autoimmune diseases remains uncertain, it is widely acknowledged that the dysregulation of adaptive immunity and the imbalance between T helper 1 and 2 cell responses are fundamental factors in the progression of autoimmune processes.

Considering that CD is characterized by inflammation occurring in the small intestine, it is reasonable to propose that the local micro-environment, which is significantly influenced by the microbiota, plays a crucial role in the disease’s pathogenesis and the breakdown of tolerance to dietary gluten. The involvement of gut microbes in the development of CD has been demonstrated through various mechanisms. The microbiota, due to its secreted peptidases, can not only form immunogenic peptides, but also, on the contrary, eliminate immunogenic peptides that are not cleaved by intestinal enzymes. Certain bacteria express epitopes resembling gliadin, thereby triggering an immune response in the host [35]. Other bacteria, such as Pseudomonas aeruginosa, can synergistically exacerbate mucosal inflammation when combined with gluten [36]. Viral enterocolitis can activate an immune response via activation of toll-like receptor (TLR) 3 [37]. Concurrently, the microbiota can influence the digestion process, generating either immunogenic or tolerogenic gluten peptides, thereby impacting antigen formation. Additionally, the microbiome can directly affect intestinal permeability by releasing zonulin and promoting the maturation of the epithelial mucosa. Gut microbes also modulate the immune system by producing peptides, metabolites, and cytokines that possess either proinflammatory or anti-inflammatory properties [38].

The development of autoimmunity in CD arises from the coexistence of genetic susceptibility and exposure to gluten, coupled with the impairment of intestinal barrier function. This leads to a proinflammatory innate immune response triggered by gluten, an insufficient adaptive immune response, and an imbalance in the gut microbiome [6].

In CD, partially digested fragments of gliadin interact with chemokine receptor 3 on the apical side of the epithelium [17], leading to the release of zonulin through a myeloid-differentiation-primary-response-88-dependent mechanism [6]. Zonulin then interacts with the intestinal epithelium, triggering an increase in intestinal permeability [39]. The compromised gut barrier allows gliadin peptides to translocate from the lumen to the lamina propria [9]. Upon translocation, gliadin peptides stimulate the release of interleukin-15, keratinocyte growth factor, and interleukin-8 [10], which results in the recruitment of neutrophils to the lamina propria [11]. Additionally, alpha-amylase/trypsin inhibitors engage the toll-like receptor 4–MD2-CD14 complex, leading to the upregulation of maturation markers and the release of proinflammatory cytokines [12]. Subsequently, innate immune-mediated apoptosis of intestinal cells occurs, resulting in the release of intracellular tissue transglutaminase. Gliadin peptides are partially deamidated during this process [13]. Deamidated gliadin is recognized by antigen-presenting cells expressing DQ2/8+ molecules [14], which then present the gliadin peptides to T helper cells [15]. T helper cells activate and mature B cells, leading to the production of IgM, IgG, and IgA antibodies against tissue transglutaminase [16]. Additionally, T helper cells produce proinflammatory cytokines such as interferon γ and tumor necrosis factor α [32], which further contribute to increased gut permeability and, in conjunction with T killer cells, initiate the development of enteropathy.

Damaged enterocytes express the CD71 transporter on their apical side, resulting in the retrotranscytosis of secretory IgA–gliadin complexes [33]. This process enhances the trafficking of gluten from the gut lumen to the lamina propria. Subsequently, the interaction between CD4+ T cells in the lamina propria and gliadin induces the activation and proliferation of these T cells. As a result, proinflammatory cytokines, metalloproteases, and keratinocyte growth factors are produced by stromal cells, which leads to crypt hyperplasia and villous blunting. Additionally, intraepithelial lymphocytes contribute to the death of intestinal epithelial cells. The hyperplastic crypts are characterized by an expansion of the immature progenitor cell compartment (WNT pathway) and downregulation of the Hedgehog signaling cascade [34]. Furthermore, an increased number of stromal cells within the intestinal stem cell niche and elevated levels of bone morphogenetic protein antagonists, such as Gremlin-1 and Gremlin-2, may further contribute to the observed crypt hyperplasia in individuals with CD.

## 5. Intestinal Microbiota—General Aspects

The term “gut microbiota” refers to all the microorganisms and their collective genome (called the “microbiome”) that we find along the entire length of the tract. Their density is so high that they are approximately equal in number to human cells [40,41]. The gastrointestinal system contains over 100 trillion microorganisms. More than 2 million genes are expressed by the human microbiome, producing thousands of metabolites. The human genome is composed of only 23,000 genes. The coexistence of organisms and microbiota results in a “superorganism” with a mutable immune and metabolic profile [42].

Children at age one are colonized by *Akkermansia muciniphila*, Bacteroides, *Veillonella*, *Clostridium coccoides* spp., and *Clostridium botulinum* spp. After the age of 3 years, the microbiota becomes relatively stable and resembles the adult microbiota, dominated by three bacterial phyla: Firmicutes (*Lachnospiraceae* and *Ruminococcaceae*), Bacteroidetes (*Bacteroidaceae*, *Prevotellaceae*, and *Rikenellaceae*) and Actinobacteria (*Bifidobacteriaceae* and *Coriobacteriaceae*) [43].

Over 90% of the intestinal microbiota is represented by Firmicutes and Bacteroidetes. The Firmicutes phylum is composed of 200 different genera, 95% of which are Clostridia. Bacteroidetes consist of Bacteroides and Prevotella [44]. 

Table 3 describes the taxonomic composition of the intestinal microbiota. Firmicutes and Bacteroidetes represent 90% of the whole gut microbiota.

Gluten is not completely digested in the intestine. Undegraded gliadin is transported from the small intestine to the large intestine. Some intestinal microorganisms such as the genera *Lactobacillus*, *Streptococcus*, *Staphylococcus*, *Clostridium*, and *Bifidobacterium* have the ability to metabolize gliadin [45].

*Bifidobacteria*, *Firmicutes*, *Lactobacilli*, and *Streptococceae*, the flora with a protective effect, are numerically reduced in BC, instead the proliferation of Gram-negative bacteria (*Bacteroides*, *Bcterioidetes*, *Bacteroides fragilis*, *Prevotella*, *E. coli*, *Proteobacteria*, *Haemophilus*, *Serratia*, *Klebsiella*) was observed [41,46]. Moreover, there are significantly increased populations of rod-shaped bacteria (*Clostridium* spp., *Prevotella* spp., and *Actinomyces* spp.) in CD patients. In the studied groups, the majority of duodenal biopsies from CD patients, compared to healthy subjects, showed dysbiosis and revealed an increased number of Gram-negative bacteria, *Bacteroides*, *Firmicutes*, *E. coli*, *Enterobacteriaceae*, and *Staphylococcus*, and a decrease in *Bifidobacterium*, *Streptococcus*, *Provetella*, and *Lactobacillu* spp. [7]. Other studies have reported a higher abundance of the phylum *Proteobacteria* (family *Enterobacteriaceae*), the genera *Bacteroides*, and *Staphylococcus* in untreated CD compared to healthy subjects [8].

In conclusion, most of the current literature has emphasized that dysbiosis in CD is marked by an increase in Gram-negative species and *Bacteroidetes* and a decrease in Bifidobacteria and Lactobacilli [47].

## 6. Overview of CD Genetics and the Risk of Developing CD in Genetically Susceptible Individuals According to Their Microbiota Pattern

The strong evidence of high familial recurrence (10–15%) and high concordance in monozygotic twins (75–80%) clearly indicates that CD, like other autoimmune diseases, has a significant hereditary component [48]. Similar to other autoimmune conditions, the HLA class II heterodimers, particularly DQ2 and DQ8, play a crucial role in disease susceptibility. Homozygosity for HLA-DQ2 carries a much higher risk (25–30%) of developing early onset CD in infants with a first-degree family member affected by the disease (16–18%). Since HLA-DQ2/HLA-DQ8 is relatively common in the general population (25–35%), and only 3% of individuals with these HLA types will actually develop the disease [49], it is understandable that genome-wide association studies have identified over 100 non-HLA genes associated with CD [48,50]. While these genes may not have a significant impact on genetic risk, they may contribute to the understanding of yet unknown mechanisms underlying the occurrence of the disease.

Olivares et al. [51] demonstrated a connection between the HLA-DQ genotype and differences in early gut microbiota. Using specific sequencing techniques, they observed that breastfed infants with a genetic predisposition to CD had a lower abundance of Bifidobacterium bacilli in their feces. Additionally, these at-risk infants showed increased numbers of Proteobacteria, as well as strains from the *Enterobacteriaceae* family [51]. The decrease in Bifidobacterium bacilli has been previously observed in other autoimmune conditions, such as rheumatoid arthritis, suggesting a potential protective role of this bacterium against inflammation [52]. This finding supports the notion that the composition of gut microbiota may influence the development of autoimmune disorders.

Studies have linked Bacteroides species, a type of commensal gut microbe, to gut inflammation, including inflammatory bowel disease [53]. In the case of infants at genetic risk for CD, Sanchez et al. [54] conducted a specific assessment of Bacteroides species using PCR and denaturing gradient gel electrophoresis. They found that infants at high genetic risk had a higher prevalence of *B. vultagus*, while infants at low genetic risk showed a higher prevalence of *B. ovatus*, *B. plebeius*, and *B. uniformis* [54]. Similarly, in a longitudinal study utilizing FISH technology, De Palma et al. [55] observed a higher proportion of Bacteroides–Prevotella in high-risk infants. Additionally, they found a significantly greater abundance of various bacteria including Gram-negative bacteria, *E. coli*, Streptococcus–Lactococcus, *E. rectale*–*C. coccoides*, sulfate-reducing bacteria, *C. lituseburense*, and *C. histolyticum* [55]. These findings suggest a potential association between the composition of Bacteroides species and the risk of developing CD in genetically susceptible individuals.

Several prospective cohort studies have investigated the dynamics of gut microbiota in infants genetically susceptible to CD [56,57]. In one such study, conducted on participants from the PROFICEL cohort in Spain [56], which followed infants at genetic risk of developing CD over 5 years, researchers compared the stool samples of infants who were fed differently (breastfed or formula-fed). The study analyzed samples collected at 7 days, 1 month, and 4 months of age and found a higher prevalence of enterotoxigenic Escherichia coli (ETEC) in genetically susceptible infants [56]. This suggests a potential association between genetic susceptibility to CD and the presence of ETEC in the gut microbiota of infants.

In a study by Sellitto et al. [57], 34 breastfed infants with a genetic risk for developing CD were monitored, and their stool samples were collected over the first 2 years of life. The study employed 16S sequencing to analyze the microbiota composition. The findings revealed that infants with high-risk genetics exhibited an increased prevalence of *Firmicutes* and *Proteobacteria*, along with an overall decreased prevalence of Actinobacteria and Bacteroidetes. Interestingly, it was observed that the stool microbiota of these genetically at-risk infants did not reach a stable state until 12 or 24 months of age, which contrasted with previous findings in healthy infants where the microbiota stabilized earlier [57]. These results highlight the distinct microbial dynamics and composition in genetically susceptible infants and suggest that the development of the gut microbiota may be influenced by genetic factors related to CD risk.

The CDGEMM study (Celiac Disease Genomic, Environmental, Microbiome, and Metabolomic Study) is a prospective longitudinal study that aims to enroll 500 infants from birth [58]. This international study, conducted in the United States, Italy, and Spain, seeks to investigate the role of gut microbiota and metabolome in the development of CD. By combining environmental, genetic, and biological data from participants, the study aims to identify risk factors associated with the onset of this condition [58,59]. In a recent study by the CDGEMM investigative team [60], metagenomic sequencing was employed to compare stool samples from birth, 3 months, and 4–6 months in infants with both standard and high genetic risk for CD. The analysis revealed an increased abundance of Bacteroides and Enterococcus in infants at both standard and high risk of CD. Furthermore, compared to individuals without disease risk, the microbiota of high- and standard-risk infants exhibited reduced numbers of *Streptococcus*, *Coprococcus*, *Veillonella*, *Parabacteroides*, and *C. perfringens* species [60]. These findings are surprising and contrary to the results of previous microbiome studies in other autoimmune conditions such as autoimmune hepatitis and Behcet’s disease, where higher numbers of *Veillonella* and *Clostridium* were observed [61,62].

## 7. Implications of Intestinal Microbiota Alterations among Celiac Disease Patients

As shown previously, infants with HLA-DQ2 and HLA-DQ8 have increased *Firmicutes* and *Proteobacteria* and less *Actinobacteria* and *Bifidobacterium*, suggesting that specific bacteria are associated with the HLA genotype [7]. The presentation of gliadin peptides triggers an inflammatory process resulting in proinflammatory cytokines (IFN-γ) and autoantibodies [63]. In studies focusing on the intestinal microbiome, some bacteria have been associated with CD in the absence of classic HLA risk alleles; the involvement of non-HLA genes is still unknown [64]. *There are two different essential situations:* in CD, there is an imbalance of intestinal microecology on one side, caused by the disease itself and, on the other side, an abnormal intestinal flora induced by several factors acting as a co-factor of gliadin in inducing the disease [45].

**Figure 1:**
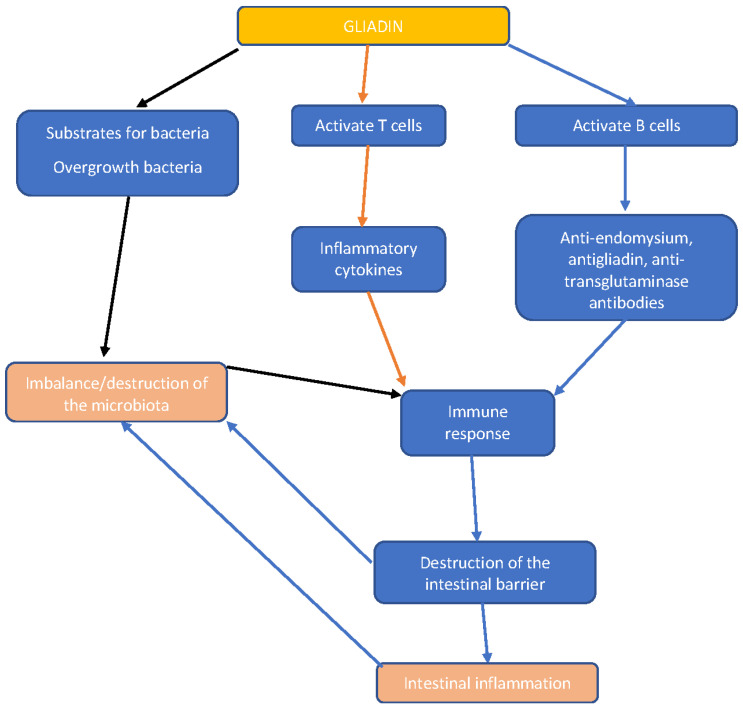
Immune response and the mechanism of inflammation. The relationship between intestinal flora and gluten in celiac disease.

Figure 1 shows the immune response and the mechanism of gut inflammation.

### 7.1. Possible Enviromental Causes of Intestinal Microbiota Alteration

#### 7.1.1. Birth Gestational Age

In premature children, colonization disorders are basically caused by organic immaturity and environmental factors, such as prolonged hospitalization (Intensive Care Unit wards), and the use of antibiotics. In these cases, the predominant family *Enterobacteriaceae*—*Proteobacteria* and the reduction in *Bifidobacterium*, *Bacteroides*, and *Atopobium* were observed. Due to the predominant enteral feeding and the lack of natural feeding at the breast, these infants are deficient in lactoferrin, which favors intestinal colonization of infants with beneficial bacteria [43].

#### 7.1.2. Type of Delivery

Cesarean delivery increases the risk of CD because the child is spared perinatal colonization, which represents the first exposure to microorganisms in the gut [65]. Dysregulation of this microbiota influences the intestinal immune response, as well as the defense/barrier function of the mucosa, thus allowing the passage of gliadin peptides through the intestinal epithelium, which is a key element in the pathogenesis of CD [66].

#### 7.1.3. Methods of Milk Feeding

As we said above, feeding and the composition of the feeding are some of the defining factors of the microbiota. The type of milk—mother’s milk or formula—represents an important factor; breast milk favors the development of Bifidobacterium spp., with their beneficial effects. It has been observed that breastfeeding at the time of introducing gluten reduces the risk of developing CD or delays its onset. It has also been observed that mothers with CD show a decrease in several immune markers, including IL-12p70, transforming growth factor (TGF)-1, and secretory IgA (sIgA), and the number of Bifidobacterium spp. in breast milk compared to healthy mothers [66].

#### 7.1.4. Body Mass Index (BMI) Classes and Exercise Frequency

Related to the children’s diet and in addition to what has been previously mentioned, it has been observed that species from the Proteobacteria genus, a Gram-negative phylum that includes species such as Rickettsia, Neisseria, and Escherichia, proliferate in obese children [43].

#### 7.1.5. Antibiotics

It is known that antibiotics destroy the intestinal microbial flora, especially when administered in the first years of life when this flora is constantly changing both in diversity and density. In both the short and long term, many studies report that antibiotics tend to favor Bacteroides enterotypes [67].

#### 7.1.6. Infections

Studying the most common infections in the first 2 years of life (Rotavirus, Enterovirus, Adenovirus type 12, Orthoreovirus, and Candida albicans), it was observed that they lead to an increased risk of developing CD. Adenovirus type 12 increases the risk of CD due to structural similarity to an amino acid sequence of gliadin. Orthoreovirus induces inadequate immune stimulation, resulting in loss of tolerance to gliadin and increased intestinal inflammation and permeability. *Candida albicans* expresses a structurally similar gliadin protein-1 and has been proposed as a possible trigger for CD [68].

Considering the above, we can deduce a multifactorial etiology of CD, the intestinal microbiota is very important, and the general consensus suggesting an association of CD with the over-representation of pathogenic bacteria and a decrease in the number of symbiotic and/or commensal bacteria [8].

#### 7.1.7. Cause or Effect?

This is the question to which an answer is desired. Are changes in the composition and function of the microbiota in CD patients the triggering cause or are they part of the changes induced by the disease? Both patients and doctors expect that, in the future, these microbiota/microbial strains that lead to gluten degradation will pave the way for a complementary CD therapy based on probiotics.

## 8. Intestinal Microbiome Modulation in Celiac Disease Patients—A New Therapeutic Perspective Besides Gluten-Free Diet

The onset of gluten intolerance may occur from the moment of its introduction in an infant’s diet or anytime later in life, showing various digestive or extra-digestive manifestations [69,70,71]. These facts launched the hypothesis that other environmental factors besides gluten ingestion are involved in the etiopathogenesis of gluten enteropathy such as gut microbiome composition, gestational age, type of birth, type of infant feeding, previous medication, or previous intestinal infections, as shown previously.

Despite acknowledging the impact of environmental factors on the intestinal microbiota, there is a lack of longitudinal research establishing the relationship between the gut microbiota and the onset of CD.

Several authors have proved that different roles of the component cells of the innate immune system in the lamina propria of the gut (dendritic cells, neutrophils, macrophages) are affected by microbiome changes [72,73]. An important innate immune response in the gut epithelium is induced by gliadin peptides—it is a marked expression of IL-15. Therefore, intraepithelial lymphocytes will be activated and will express the NK-G2D receptor, which is a natural killer hallmark, inducing enterocyte alterations [74,75].

The gut microbiota plays a fundamental role in regulating digestion throughout the gastrointestinal tract and has a substantial influence on the synthesis of numerous nutrients and metabolites. Additionally, the intestinal microbiota plays a vital role in immune function, as it hinders bacterial proliferation and upholds the integrity of the intestinal epithelium [76].

Recent findings endorse the notion that alterations in the composition and functionality of the gut microbiome are associated with chronic inflammatory diseases, including CD [43]. While adhering to a GFD can impact the diversity and composition of the gut microbiota, multiple investigations substantiate the theory that the microbiota is involved in the development of, clinical presentation of, and susceptibility to CD [77]. Furthermore, research has demonstrated that individuals with persistent symptoms after adhering to a long-term GFD exhibit an aberrant composition of the microbiota [78].

There is scientific evidence from various studies that suggest the early gut microbiota composition can be affected by the genotype of infants carrying the HLA-DQ2 haplotypes and at familial risk of developing CD [79]. De Palma et al. [80] conducted a study on 164 healthy neonates who had one first-degree relative with CD, which revealed that the composition of gut microbiota in infants is influenced by the HLA-DQ genotype in conjunction with the type of milk feeding.

Diet is another crucial factor in regulating the development and maintenance of gut microbiota. Even though recent studies have found that breastfeeding does not protect against the development of CD, it has been shown that the differences in microbiota composition related to genotype are reduced by breastfeeding [81]. Furthermore, human milk oligosaccharides improve the overall barrier function of the gut by reducing the susceptibility of enterocytes to bacterial-induced innate immunity [82].

Changes in the composition of the gut microbiota can contribute to the alteration of the intestinal barrier and an increase in the permeability of the epithelial layer [83]. Disruption of the protein called zonulin, which is an important component of tight junctions, is responsible for the increased intestinal permeability observed in patients with CD. According to several studies, dysbiosis has been linked to elevated zonulin release, resulting in the breakdown of tight junctions and facilitating the penetration of partially broken-down peptides of gliadin into the lamina propria [84].

Several cross-sectional studies have been conducted in recent years to investigate the salivary, fecal, and duodenal microbiota in patients with CD. These studies have found that patients with CD have lower levels of beneficial species such as *Lactobacillus* and *Bifidobacterium* and higher levels of potentially pathogenic species such as *Bacteroides* and *E. coli*, compared to healthy individuals [85].

A study compared the effects of Bifidobacterium longum and *Bifidobacterium bifidum* on peripheral blood mononuclear cells with those of Gram-negative bacteria, such as *Escherichia coli* and *Bacteroides fragilis*, either alone or in combination with CD triggers. It was found that Gram-negative bacteria induced a higher secretion of TH-1 proinflammatory cytokines and activation mechanisms (CD40, IL-12, HLA-DR, and IFN-c) than the Bifidobacterium strains [8,86].

In the human colon, the gut microbiota is involved in gluten metabolism, and certain strains such as *Bifidobacterium* spp. and *Lactobacilli* may have a role in breaking down gluten and its peptides to alter immunogenicity [87,88].

Studies conducted in vitro have shown that certain strains of *Lactobacilli*, when incorporated into the sourdough fermentation process, break down gluten peptides rich in proline and glutamine, resulting in a gluten concentration of fewer than 10 parts per million (gluten-free) and reduced immunotoxicity. Furthermore, during simulated gastrointestinal digestion, Lactobacilli strains from a pooled probiotic culture have been observed to hydrolyze synthetic peptides rich in proline that are implicated in CD [7].

From the upper gastrointestinal tract of pigs, four strains of *Lactobacilli* (*L. salivaris*, *L. ruminis*, *L. amylovorus*, and *L. Johndoni*) with the most significant activity in degrading gliadin peptides, which decreases their ability to induce CD, were isolated and identified in vitro [89].

In a study that included 20 individuals diagnosed with CD who consumed *L. hilgardi*, *L. alimentaris*, *L. sanfranciscenis*, and *L. brevis* containing hydrolyzed wheat gluten bread for six days, there was no noteworthy rise in interferon-γ (INF-γ) secretion in comparison to the healthy control group [90]. In vivo studies were also conducted on CD patients in remission, who were challenged with Lactobacilli-predigested gluten for 60 days. The outcomes were promising since there was no deterioration in serological indicators, symptoms, or intestinal permeability, implying that the Lactobacilli-derived endopeptidase was effective in completely breaking down gluten and minimizing its detrimental effects in patients with CD [91].

Caminero et al. [36] found that both pathogenic microorganisms and essential gut commensals have varying abilities to break down gluten into diverse immunogenic patterns, which could affect the risk of autoimmunity. They specifically observed that *Lactobacilli* can eliminate gliadin peptides after they are partially digested by human proteases and that the immunogenic peptides generated by Pseudomonas aeruginosa proteases lose their immunogenicity in the presence of Lactobacillus. These findings indicate that specific microbial strains could potentially serve as probiotics in the adjunctive therapy of CD.

Furthermore, a recent investigation has demonstrated the role of gut microbiota and their metabolites in increasing susceptibility to autoimmunity through epigenetic mechanisms [92]. In the search for a microbial agent to modulate disease, *Bifidobacteria* and *Lactobacilli* are the most extensively studied strains. *Bifidobacteria* strains have been found to reduce the epithelial permeability that is induced by gluten [93], to decrease the typical Th1 pathway activation seen in CD [94], and to decrease damage to the jejunal architecture [95]. Additionally, research has reported that *Escherichia coli* may have a protective impact on gut barrier function [96], and specific strains of *Lactobacilli* have been found to possess immunomodulatory characteristics [97].

Moreover, research suggests that the makeup of the gut microbiota can impact the permeability of the intestinal lining [98], and probiotics have been found to promote the production of short-chain fatty acids (SCFAs), specifically butyrate, which can effectively modulate proinflammatory activities within the colon and have beneficial effects on the health of the colon’s epithelial cells [99].

Given the known involvement of microbiota in gluten processing and immune response modulation, utilizing probiotics to manipulate the microbiome presents a novel approach to address CD and its associated symptoms.

In current medical practice, a strict GFD is the only effective treatment available for CD. This approach can bring about the resolution of both intestinal and extraintestinal symptoms, along with the re-growth of the intestinal villi and the negativity of autoantibodies. Furthermore, a GFD offers partial protection against several complications. However, this treatment has some drawbacks that should be taken into account. For example, a GFD can have a negative impact on quality of life, leading to psychological problems and fear of inadvertent contamination with gluten. Multicenter studies focusing on gluten immunogenic peptides (GIP) [6,100] have demonstrated the prevalence of such fears among patients. Other disadvantages include an elevated risk of cardiovascular disorders, metabolic syndrome, severe constipation, and possible deficiencies in vitamins and minerals [101,102,103]. Instructing CD patients about the risks of an uncontrolled GFD and providing nutritional recommendations by an experienced dietitian can help overcome most of the drawbacks associated with the disease. Additionally, psychological support from a psychologist can be highly beneficial in helping patients accept the disease [104]. Because gluten withdrawal can lead to a significant decrease in quality of life, nearly 40% of individuals with CD are unsatisfied with their dietary restrictions and are interested in investigating alternative therapies [105]. In the past few years, researchers have been working to fulfill the demands of individuals with CD who are seeking alternative treatments beyond standard diet therapy [106].

Studies of probiotics’ effects on CD conducted on animal models have demonstrated potential beneficial effects. In particular, *Bifidobacterium longum* CECT 7347 has been found to reduce the production of CD 4+ T cells and inflammatory cytokines in rats [95] and also to improve gliadin-induced enteropathy [88]. Oral administration of Saccharomyces boulardii KK1 was found to reduce the expression of epithelial cell CD71 and local cytokine production and to improve enteropathy in mice sensitized to gluten [89]. In a mouse model of gliadin-induced villous damage, administration of Lactobacillus casei was found to be effective in restoring the normal mucosal architecture [97]. Bifidobacterium breve has been reported to prevent intestinal inflammation by promoting the production of intestinal IL-10-producing Th1 cells [107]. In mouse models, it has also been found to improve symptoms of dextran sulfate sodium (DSS)-induced colitis and modulate T cell polarization toward Th2 and Tregs, both in vitro and in vivo [108]. In a recent investigation conducted by Orlando et al., it was observed that the administration of *Lactobacillus rhamnosus* GG to rats could protect the intestinal mucosa from damage caused by gliadin peptides [109].

Although in vitro and animal studies have shown promising results in the use of probiotics, there is still a lack of homogeneous data from human trials. There are only a few studies on the use of probiotics as an intervention for CD patients on a GFD. Nonetheless, these limited results suggest that combining probiotics with a GFD can assist in the recovery of the intestinal microbiota in patients with CD. According to studies, children with CD show a decrease in the abundance of *Actinobacteria* and lower ratios of Firmicutes to Bacteroides than healthy control.

In the context of CD, the utilization of probiotics can potentially influence the microbiota’s composition and functions, thereby preventing or delaying the disease onset. Probiotics can regulate various processes such as toxin receptor degradation, adhesion site blockage, production of inhibitory substances against pathogens, and nutrient competition immune response [110].

We conducted a literature review of studies on human patients with CD published between January 2000 and December 2022, examining the effectiveness and safety of probiotic supplementation, and the findings are summarized in Table 4. While few adverse effects were reported in CD patients treated with probiotics, a limited number of studies suggest that probiotic supplementation may improve symptoms related to CD.

It is common for patients with CD who adhere to a GFD to experience persistent symptoms. Probiotics, particularly those related to gliadin metabolisms such as Bifidobacterium and Lactobacillus, are expected to serve as adjuvant therapy for CD patients, potentially reducing adverse reactions associated with a strict GFD. While probiotics have shown promise in managing symptoms in CD patients on a GFD, the available data are limited and therefore not conclusive.

Prebiotics have emerged as a promising and safe addition to the GFD among new therapies proposed recently, with a positive impact on human health [126]. By stimulating the growth and activity of beneficial bacterial strains such as Bifidobacterium and Lactobacillus in the gut, prebiotics can regulate gut microbiota and potentially alleviate symptoms related to CD. Based on evidence from the literature, it is hypothesized that adding prebiotics to the GFD could be an economical and convenient adjunctive therapy for CD [126]. To date, there have been only a limited number of preliminary human studies investigating the effects of prebiotics on intestinal inflammation in general and CD in particular [114,127]. One of the initial investigations on this topic was conducted by Krupa-Kozak et al. [127], who performed a randomized placebo-controlled clinical trial to evaluate the impact of oligofructose-enriched inulin called “Orafti^®^-Synergy1” (Tienen, Belgium) on children with CD who were following a GFD. In their analysis of the pediatric population, the researchers observed a reduction in Lactobacillus counts and a rise in Bifidobacterium. Meanwhile, Adebola et al. [128] revealed that inulin did not have a direct stimulating effect on any of the five probiotic strains of Lactobacillus. However, other prebiotics such as lactulose and lactobionic acid may have this effect and provide an ideal substrate for bacteria to mitigate the negative impacts of bile acid stress. Tuohy et al. [129] conducted a comparable investigation where they noted a notable rise in Bifidobacterium numbers among healthy participants who took inulin for two weeks. Additionally, a crucial study [127] revealed that incorporating oligofructose-enriched inulin into the GFD enhanced fecal microbiota and substantially increased total SCFAs such as propionate and butyrate.

## 9. Conclusions

For the moment, the only treatment available in current practice for CD patients worldwide is a GFD. Undiagnosed or noncompliant patients are exposed to the risk of long-term complications such as anemia, infertility, osteoporosis, or cancer, especially intestinal lymphoma. In the coming years, identifying other target genes and understanding the pathways they influence will lead to a better understanding of CD pathogenesis. Ultimately, we might be able to define genetic risk profiles for more precise diagnoses and for predicting disease progression, leading to novel therapies.

In recent research, various dietary interventions have been tested in order to optimize the response to a GFD and to increase the compliance of celiac patients. The modulation of gut–intestinal microbiota could represent a beneficial therapeutic strategy. Preliminary results proved that adding prebiotic and probiotic supplements after gluten exclusion could decrease intestinal hyperpermeability and improve the gut immune response, restoring normal villous architecture. Still, larger randomized controlled trials should be performed to sustain the role of pre- and probiotics administration in CD patients. An essential requirement of future studies would be to determine the type of probiotic to be administered, the dose, and the period of administration.

## Figures and Tables

**Table 1:** Serological screening for celiac disease in adults (confirmed with duodenal biopsy) in the general population.

Author	Country	Age, Years	First-Level Antibody Test	Prevalence of Celiac Disease
Corazza et al., 1997 [9]	Italy	20–87	EmA	0.18%
Ivarsson et al., 1999 [10]	Sweden	25–74	EmA	0.53%
Riestra et al., 2000 [11]	Spain	14–89	EmA	0.26%
Volta et al., 2001 [12]	Italy	14–65	EmA	0.57%
Mustalahti et al., 2010 [13]	Finland	30–93	Anti-tTG, EmA	2.5%
Rubio-Tapia et al., 2012 [14]	USA	23–66	Anti-tTG, EmA	0.71%
Singh et al., 2016 [15]	Asia	Not specified	Anti-tTG, EmA	0.5%

Anti-tTg: anti-transglutaminase antibodies; EmA: anti-endomysium antibodies.

**Table 2:** The global prevalence of celiac disease.

	Seroprevalence	Biopsy Prevalence
Global	1.4% (95% CI 1.1–1.7)	0.7% (95% CI 0.5–0.9)
Africa	1.1% (95% CI 0.4–2.2)	0.5% (95% CI 0.2–0.9)
Asia	1.8% (95% CI 1–2.9)	0.6% (95% CI 0.4–0.8)
Europe	1.3% (95% CI 1.1–1.5)	0.8% (95% CI 0.6–1.1)
Oceania	1.4% (95% CI 1.1–1.8)	0.8% (95% CI 0.2–1.7)
North America	1.4% (95% CI 0.7–2.2)	0.5%
South America	1.3% (95% CI 0.5–2.5)	0.4% (95% CI 0.1–0.6)

Singh et al. [30]. CI: confidence interval.

**Table 3:** Taxonomic composition of the intestinal microbiota.

Phylum	Class	Order	Family	Genus	Species
*Actinobacteria*	*Actinobacteria*	*Actinomycetales*	*Corynebacteriaceae*	*Corynebacterium*	
		*Bifidobacteriales*	*Bifidobacteriaceae*	*Bifidobacterium*	*B. longum*
					*B. bifidum*
	*Coriobacteriia*	*Coriobacteriales*	*Coriobacteriaceae*	*Atoopobium*	
*Firmicutes*	*Clostridia*	*Clostridiales*	*Clostridiaceae*	*Faecalibacterium*	*F. prausnitzii*
				*Clostridium*	*Clostridium spp.*
			*Lachnospiraceae*	*Roseburia*	*R. intestinalis*
			*Ruminococcaceae*	*Ruminococcus*	*R. faecis*
		*Veillonellales*	*Veillonellaceae*	*Dialister*	*D. invisus*
	*Negativicutes*	*Lactobacillales*	*Lactobacillaceae*	*Lactobacillus*	*L. reuteri*
	*Bacilli*		*Enterococcaceae*	*Enterococcus*	*E. faecium*
		*Bacillales*	*Staphylocoecaceae*	*Staphylococcus*	*S. leei*
*Bacteroidetes*	*Sphingobacteriia*	*Sphingobacteriales*	*Sphingobacteriaceae*	*Sphingobacterium*	
	*Bacteroidia*	*Bacteroidales*	*Bacteroidaceae*	*Bacteroides*	*B. vulgatus*
					*B. fragilis*
					*B. uniformis*
			*Tannerellaceae*	*Tannerella*	
				*Parabacteroides*	*P. distasonis*
			*Rikenellaceae*	*Alistipes*	*A. finegoldi*
			*Prevotellaceae*	*Prevotella*	*Prevotella spp.*
*Proteobacteria*	*Delta Proteobacteria*	*Enterobacterales*	*Enterobacter*	*Escherichia*	*E. coli*
				*Shigella*	*S. flexneri*
		*Desulfovibrionales*	*Desulfovibrionaceae*	*Desulfovibrio*	*D. intestinalis*
				*Bilophilia*	*B. wadsorthia*
	*Epsilon Proteobacteria*	*Campylobacterales*	*Helicobacteraceae*	*Helicobacter*	*H. pylori*
*Fusobacteria*	*Fusobacteriia*	*Fusobacteriales*	*Fusobacteriaceae*	*Fusobacterium*	*F. nucleatum*
*Verrucomicrobia*	*Verrucomicrobiae*	*Verrucomicrobiaales*	*Akkermansiaceae*	*Akkermansia*	*A.muciniphila*

**Table 4:** Papers assessing the beneficial role of probiotic administration in CD animal models and human subjects.

Author	Year	Probiotic Variety	Objectives and Discoveries	Conclusions
De Angelis et al. [111]	2006	VSL#3	VSL#3 exhibits a significant capacity for long-term colonization of the intestine.	Administration of VSL#3 would result in the complete eradication of toxic peptides in processed foods, thereby reducing long-term health risks and enhancing overall quality of life.
Medina M. et al. [112]	2007	*Bifidobacterium longum*	The genomic DNA of certain strains elicited a Th1 and proinflammatory cytokine response, characterized by the production of interferon-gamma and TNF-α, rather than IL-10.	The capacity of *B. longum* to modulate the immune system.
Lindfors K. et al. [93]	2008	*Bifidobacterium lactis*	Prevent the harmful effects on the mucous membrane of the small intestine induced by gluten/gliadin.	The inhibition is dose-dependent and results in increased permeability of epithelial cells induced by gliadin. Additionally, it stimulates the production of IL-10 by regulatory T cells.
D’Arienzo et al. [97]	2011	*Lactobacillus casei* ATCC 9595	There was a full recovery of the blunting of villi, decreased weight loss, and the basal levels of TNF-α were restored.	The use of *L. casei* was successful in restoring the normal structure of the mucosal lining and maintaining homeostasis in the gut-associated lymphoid tissue.
Papista et al. [113]	2012	*Saccharomyces boulardii* KK1 strain, hydrolyzed the 28 kDa gliadin fraction	Administration *of S. boulardii* improved the development of enteropathy, reduced expression of CD71 in epithelial cells, and limited the production of cytokines in localized areas.	A novel mouse model has been developed for studying human CD that shares histopathological features and common biomarkers. The treatment of CD using *S. boulardii* was found to be effective in reversing the development of the disease.
Laparra et al. [95]	2012	*Bifidobacterium longum* CECT 7347	In animals with gluten sensitivity, the administration of *B. longum* resulted in increased expression of NF-κB, IL-10, and CD8+ cells but reduced expression of TNF-α, CD4+ cells, and CD4+/Fox3+ cell populations.	In an animal model of gliadin-induced enteropathy, *B. longum* modulates the production of inflammatory cytokines and the immune response mediated by CD4+ T cells.
Smecuol et al. [114]	2013	*Bifidobacterium natren* *life start*	The impact of gluten on intestinal permeability, clinical symptomatology assessed through the GSRS questionnaire, and changes in immunological markers.	Supplementation of untreated CD patients with *Bifidobacterium NLS* did not alter protein abnormalities but demonstrated potential for symptom improvement and elicited immunological changes.
Golfetto et al. [115]	2014	*Bifidobacteria spp.*	The quantity of *Bifidobacteria* per gram of fecal matter was markedly greater in healthy controls (1.5 ± 0.63 × 10^8^ CFU/g) in comparison to celiac patients (2.5 ± 1.5 × 10^7^ CFU/g).	Diminished levels of *Bifidobacteria* may disrupt the intestinal microbiota equilibrium in individuals with CD, irrespective of pH and adherence to a GFD.
Pisarello et al. [116]	2014	*Lactobacillus rhamnosus;* *Lactobacillus paracasei*	The group of children with CD following a GFD exhibited markedly lower *Lactobacillus* counts compared to the healthy control group.	Probiotic therapy is not a substitute for a GFD, but it has the potential to mitigate the aberrant inflammatory parameters observed in individuals with CD, as well as modulate the composition of the intestinal microbiota.
Olivares et al. [117]	2014	*Bifidobacterium longum* CECT 7347	Measures of immune phenotype in peripheral blood cells, serum cytokine levels, fecal secretory IgA, anthropometric parameters, and composition of intestinal microbiota at both baseline and following intervention.	Patients undergoing probiotic therapy exhibited an increase in height percentile, a reduction in peripheral CD3+ T lymphocytes, and a slight decrease in TNF-α concentration. Furthermore, decreased levels of *B. fragilis* and secretory IgA were observed in the stool.
Klemenak et al. [118]	2015	*Bifidobacterium breve* BRO3 and *B. breve* B632	Results: serum levels of IL-10 and TNF- production.	Following 3 months of probiotic therapy, TNF-α levels exhibited a decrease; however, on subsequent follow-ups after another 3 months, levels showed an increase. The levels of IL-10 were below the detection threshold.
Harnett et al. [119]	2016	The De Simone formulation, previously known as VSL#3, is a blend of 9 strains of lyophilized bacteria, containing 450 billion viable microorganisms.	Quantitative analysis of microbial populations, with comparisons made between baseline and end-of-study measurements of dominant, pathogenic, and opportunistic bacteria. Evaluation of urinary metabolomics and fecal lactoferrin.	Over 12 weeks, no noteworthy alterations were observed in the gastrointestinal microbial populations of individuals with CD who exhibited persistent symptoms.
Quagliariello et al. [120]	2016	*Bifidobacterium breve* strains B632 and BRO3	Assessment of the microbiome following the probiotic intervention.	A 3-month course of probiotic therapy can result in the recovery of the microbiota of children with CD to a level similar to that of healthy individuals.
Pinto-Sanchez et al. [121]	2017	*B. infantis Natren Life Start* super strain.	Assess the mucosal expression of innate immune markers through the evaluation of the number of macrophages, Paneth cells, and α-defensin-5 expression using immunohistochemistry in duodenal biopsies.	Analysis of duodenal biopsies showed that the administration of *Bifidobacterium infantis* NLS-SS resulted in a reduction in all three innate markers in patients with CD. However, the decrease in macrophage counts was more significant in patients who followed a GFD.
Martinello et al. [122]	2017	Yogurt containing probiotic strains from PIA, Nova Petropolis-RS, with an unspecified concentration of microorganisms.	Bifidobacterial levels in fecal samples following the ingestion of 100 g of yogurt in the morning.	The fecal count of *Bifidobacteria* was greater in healthy individuals than in those with CD. While the consumption of probiotic yogurt resulted in increased *Bifidobacteria* levels in CD patients, there was no such effect observed in healthy participants.
Primec et al. [123]	2019	*Bifidobacterium breve* strains B632 and BRO3.	Assess the impact of probiotics on the composition of the fecal microbiota, levels of SCFA, and the concentration of TNF-α in the serum.	There was a strong correlation between CD and the presence of *Verrucomicrobia, Paracubacteria*, and some unidentified phyla of bacteria and archaea.
Francavilla et al. [124]	2019	A probiotic product comprising five bacterial strains: *L. casei, L.* *plantarum, B. animalis* subsp. Lacti, *B. breve* Bbr8 LMG P-17501, and *B. breve* B110 LMG P-17500.	Assess the efficacy of probiotics in improving gastrointestinal symptoms using the IBS-SSS.	Probiotic treatment resulted in a significant reduction in IBS-SSS and GSRS scores and improvement in IBS symptoms compared to placebo. Additionally, in CD patients adhering to a strict GFD, probiotics were able to modify the gut microbiota by increasing the number of *Bifidobacteria.*
Uusitalo et al. [125]	2019	*L. reuteri; L. rhamnosus,* and some unidentified.	To investigate the potential link between probiotic intake via dietary supplements or infant formula starting from one year of age and the risk of developing CD or CDA.	In general, exposure to probiotics during the first year of life was not linked to the development of CDA or CD. Nonetheless, the consumption of probiotics through dietary supplements was related to a higher risk of CDA.

Celiac disease (CD); tumor necrosis factor-alpha (TNF-α); celiac disease autoimmunity (CDA); gastrointestinal symptom rating scale (GSRS); gluten-free diet (GFD); interleukin 10 (IL-10); irritable bowel syndrome (IBS); irritative bowel syndrome severity scoring system (IBS-SSS); short-chain fatty acids (SCFA); Natren life start (NLS).

## Data Availability

The data presented in this study are available on request from the corresponding author.

## Abbreviations

anti-tTg	Anti-transglutaminase antibodies
anti-tTG2	Autoantibodies against tissue transglutaminase 2
BMI	Body mass index
CD	Celiac disease
CDA	Celiac disease autoimmunity
CDGEMM	Celiac Disease Genomic, Environmental, Microbiome, and Metabolomic Study
DSS	Dextran sulfate sodium
EmA	Anti-endomysium antibodies
ETEC	Enterotoxigenic *Escherichia coli*
*E. coli*	*Escherichia coli*
GI	Gastrointestinal
GSRS	Gastrointestinal symptom rating scale
GFD	Gluten-free diet
IBD	Inflammatory bowel disease
IBS	Irritable bowel syndrome
IBS-SSS	Irritative bowel syndrome severity scoring system
IgA	Immunoglobulin A
IL-10	Interleukin 10
INF-γ	Interferon-γ
NLS	Natren life start
NCGS	Non-celiac gluten sensitivity
sIgA	Secretory IgA
SCFAs	Short-chain fatty acids
TGF	Transforming growth factor
TNF-α	Tumor necrosis factor-alpha
TLR	Toll-like receptor
tTG	Tissue transglutaminase
T1DM	Type 1 diabetes mellitus
WA	Wheat allergy
